# *In Situ* Characterization of Hfq Bacterial Amyloid: A Fourier-Transform Infrared Spectroscopy Study

**DOI:** 10.3390/pathogens8010036

**Published:** 2019-03-18

**Authors:** David Partouche, Valeria Militello, Andrea Gomez-Zavaglia, Frank Wien, Christophe Sandt, Véronique Arluison

**Affiliations:** 1 Laboratoire Léon Brillouin LLB, CEA, CNRS UMR12, Université Paris Saclay, CEA Saclay, 91191 Gif-sur-Yvette, France; davidmeyer.partouche@gmail.com; 2 Synchrotron SOLEIL, L’Orme des Merisiers, Saint Aubin BP48, 91192 Gif-sur-Yvette, France; frank.wien@synchrotron-soleil.fr; 3 Department of Physics and Chemistry, University of Palermo, 90128 Palermo, Italy; valeria.militello@unipa.it; 4 Center for Research and Development in Food Cryotechnology (CIDCA, CCT-CONICET La Plata), RA1900 La Plata, Argentina; angoza@qui.uc.pt; 5 Université Paris Diderot, Sorbonne Paris Cité, 75013 Paris, France

**Keywords:** bacterial amyloid, functional amyloid, protein fibrils, protein fibrillation inhibition, Hfq, FTIR

## Abstract

Hfq is a bacterial protein that regulates gene expression at the post-transcriptional level in Gram-negative bacteria. We have previously shown that *Escherichia coli* Hfq protein, and more precisely its C-terminal region (CTR), self-assembles into an amyloid-like structure in vitro. In the present work, we present evidence that Hfq unambiguously forms amyloid structures also in vivo. Taking into account the role of this protein in bacterial adaptation and virulence, our work opens possibilities to target Hfq amyloid self-assembly and cell location, with important potential to block bacterial adaptation and treat infections.

## 1. Introduction

Antibiotic resistance is one of the most urgent risks to the public’s health. It occurs when bacteria develop mechanisms to defeat compounds designed to kill them [1]. Recent research for new antibiotics explores potential compounds that target proteins involved in bacterial adaptation to their environment [2]. Such adaptation allows bacteria to survive in their host and infection to progress. To achieve this goal, bacteria commonly use regulation at the post-transcriptional level [3,4]. In vivo, a protein called Hfq is often required for such a regulation. Hfq, standing for Host Factor Q**β** bacteriophage, is a bacterial protein involved in many cellular pathways, including sugar uptake, iron acquisition, bacterial virulence or pathogenicity [5,6,7,8,9,10]. Indeed, Hfq is a pleiotropic regulator that controls gene expression using noncoding RNAs (ncRNA) as cofactors [11]. ncRNAs from different species have been identified and among them, those from bacteria are ~100 nucleotides long, hence their name of small RNA (sRNA) [12,13]. Hfq-linked sRNAs function by base pairing with regions around the translation initiation signal of an associated mRNA target and therefore act on both mRNA translation and stability [14,15]. In vivo, Hfq is required for sRNA-based regulation as it promotes annealing of the regulatory sRNA to its cognate mRNA [11,16]. Hfq pleiotropic function is thus related to the diversity of sRNA-mRNA targets (see as an example multiple targets of only one sRNA, DsrA [17,18,19]). Among these targets, nearly half of sRNAs regulate the expression of membrane proteins or of proteins involved in membrane-related processes, including proteins involved in the control of virulence [20,21,22]. A part of the Hfq protein is thus needed at the periphery of the cell, and an important fraction of it is indeed located in the proximity of the bacterial inner membrane [23,24,25].

Structurally, Hfq adopts an Sm-like structure, comprising about 65 amino acids residues, which folds into a five-stranded antiparallel β-sheet capped by an N-terminal α helix [26]. The β-strands of adjacent monomers assemble into a continuous intermolecular β-sheet to form a torus [26]. Hfq differs from eukaryotic Sm proteins in that Sm proteins fold into a heptameric torus, while Hfq proteins form a 66 kDa homo-hexamer [27]. Until now, all atomic structures of *E. coli* Hfq, composed of 102 amino acids, lack its C-terminal region (CTR) composed of 38 amino acids [11].

Hfq forms organized nano-assemblies in vivo, associated with the membrane, whose cellular pattern resembles that of long-range bacterial cytoskeletal structures [24,25]. Strikingly, these nanostructures are observable only in the presence of Hfq CTR [24], an apparently intrinsically disordered fragment that spontaneously forms amyloid structures in vitro [24,28,29]. These amyloid structures form for both the full-length protein and the isolated CTR region [24,30]. Even if the role of this disordered CTR is controversial for RNA-based regulation, it has been clearly established to increase hexamer stability, to modulate DNA compaction and to affect membrane integrity [29,31,32,33,34]. Nevertheless, evidence in vivo for the formation of this bacterial amyloid structure is still lacking.

In this study, we show unambiguously that Hfq CTR forms an amyloid structure in vitro but also in vivo. Fourier transform infrared (FTIR) spectroscopy, a well-established tool for characterizing protein secondary and tertiary structure (through the amide I and amide II bands, respectively) has been used. Infrared spectroscopy indeed provides important contributions in the field of protein molecular structure and mechanisms. Main advantages of this technique are time resolution (<1 μs), and applicability from small to large protein complexes, hydrated or as dried samples. Using this technique, we already showed that Hfq-CTR has an intrinsic property to self-assemble independently of the rest of the protein [24]. In parallel, we also showed previously that the secondary structure of amyloid fibrils differs from native proteins in several conditions [35,36]. This difference, that can be seen in the amide I region of the IR-spectrum, is linked to the packing of β strands and possibly to the torsion of β sheets. Thus, the presence of amyloid fibrils is usually detected in the 1615 cm^−1^ to 1625 cm^−1^ region of the amide I band, whereas native β-sheets absorb more in the 1630 cm^−1^ and 1675 cm^−1^ regions [37,38].

## 2. Results and Discussion

### 2.1. FTIR spectra and Variance Between Hfq Mutated Strains

Taking into account the nanostructures made by Hfq in vitro and their sub-membrane clustering in the cell [24], we investigated the possibility of detecting in vivo the FTIR signal associated with Hfq β-amyloid structure. Intracellular amyloids are usually confirmed using fluorescent dyes such as Thioflavin S/T (ThT/S) or derivative on fixed bacteria [39,40]. Indeed, the use of thioflavin is a widely used method to detect the presence of amyloid fibrils in vivo or in vitro [41]. Nevertheless, we observed previously that ThT staining results in Hfq amyloid fiber disassembly [28,42]. In addition and contrast, thioflavin has been reported also to promote fibrillization [43]. The application of FTIR spectroscopy, a label-free method, may be preferable to probe in vivo the presence of Hfq amyloids. For this goal, we used a strain allowing tunable expression of various forms of the protein, namely full-length Hfq (WT), truncated Hfq without amyloid CTR (Hfq-NTR72), and a control *hfq* deletion. 

**Figure 1:**
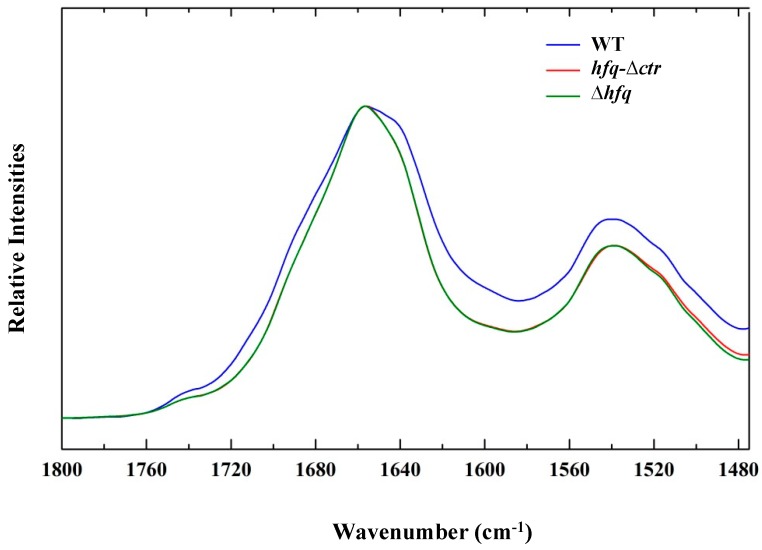
FTIR amide spectra of the three different strains WT, *hfq*∆*ctr* and ∆*hfq*.

We first used strains allowing chromosomic expression of the various forms of Hfq [29]. Nevertheless, the results were ambiguous, probably due to the presence of other amyloidogenic proteins in *E. coli* [44]. For this reason, we next used a strain allowing slight overexpression of Hfq (~5 fold) from a plasmid under the control of an inducible promoter. Note that the strain not transformed by a plasmid was totally devoid of Hfq (∆*hfq*), to avoid massive overexpression of Hfq that could be harmful to the cell [45,46]. After induction, the cells expressing Hfq are recovered by centrifugation to remove the inducer and culture media. Different plasmids were used allowing the expression of *(i)* full-length, *(ii)* a truncated form of Hfq, Hfq∆CTR (or Hfq-NTR72), and *(iii)* a control plasmid lacking Hfq. The corresponding strains will be referred through the manuscript as WT, *hfq*∆*ctr* and ∆*hfq*, respectively. As shown in Figure 1, we clearly observe a shoulder for the strain expressing the full-length Hfq protein, compared to strain expressing only the NTR72 part of Hfq (*hfq*∆*ctr)* and that of the control without Hfq (∆*hfq*), while the Amide II profile is quite similar for all samples, so we concentrate our analysis in the amide I region.

**Figure 2:**
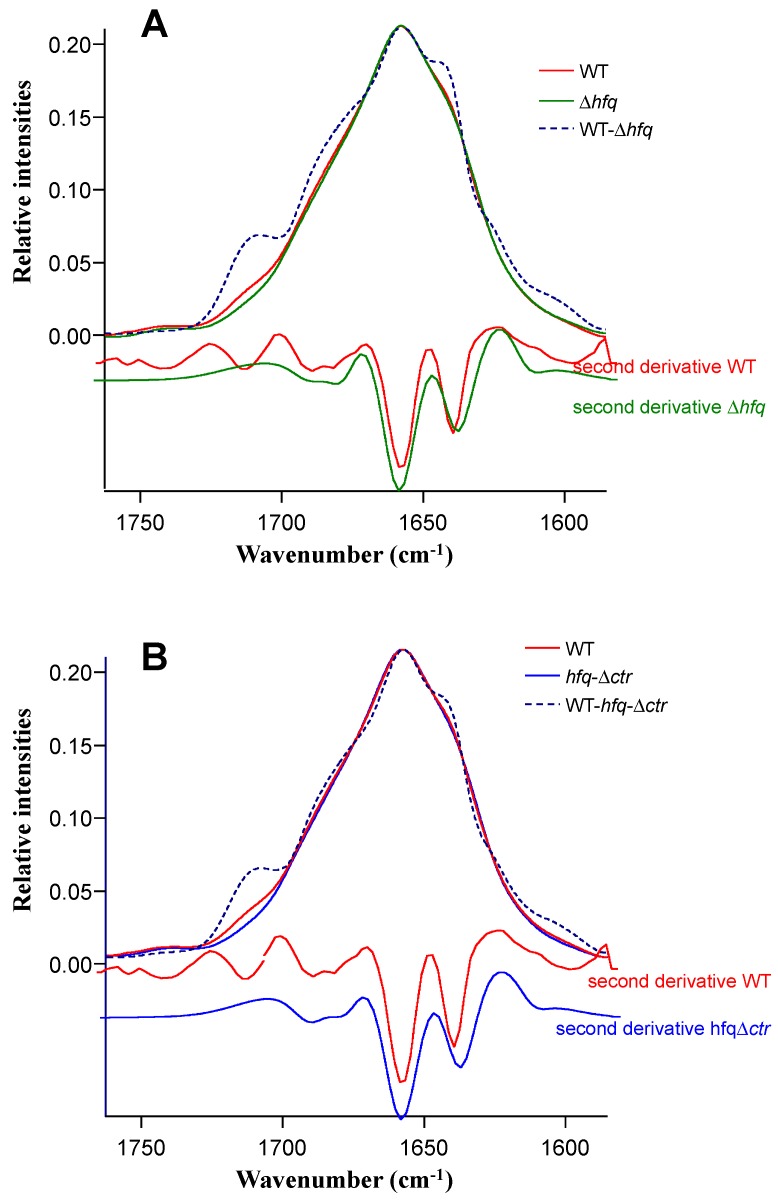
Difference spectra. (**A**) Difference spectrum WT strain − ∆*hfq* strain. (**B**) Difference spectrum WT strain − *hfq*∆*ctr* strain. Second derivative analysis of each strain is also presented.

As seen in Figure 1, no difference was observed in spectra of strains expressing NTR72 (red) and devoid of Hfq (green), while the strain expressing full-length Hfq was clearly different. Note that levels of expression of Hfq may slightly vary in different experiences, influencing the spectrum and the relative secondary structures content. To further investigate in the origin of these differences and to assign the contribution of the bands to the spectrum, we made difference spectra (Figure 2). The difference spectra between strains expressing full-length Hfq and totally devoid of Hfq (Figure 2A) and full-length Hfq and Hfq devoid on its CTR only (Figure 2B) are presented. In Figure 2A, by subtraction of *Δhfq* spectrum from WT spectrum, we have the possibility to evaluate the Amide I of Hfq-WT protein in vivo, while in Figure 2B subtraction of *hfq-∆ctr* spectrum from WT spectrum allows evaluation of the Amide I of Hfq-CTR protein in vivo. These two difference spectra demonstrate the differences in the region of the intermolecular β-sheet aggregates, the typical IR amyloid bands. The position of these amyloid peaks is close to that reported for Hfq CTR region in vitro [24].

Note that Hfq overexpression may result in a change in the expression of other proteins forming various types of secondary structure. These include unordered structures with signatures between 1640–1648 cm^−1^ and amyloid structures with signatures ~1620 cm^−1^. However, Hfq-dependent pathways usually down-regulate the expression of other proteins, as in the case of CsgD for example [47]. Indeed, Hfq-dependent sRNAs are known to repress the expression of CsgD [48]. Thus Hfq overexpression will result in a decrease in CsgD expression (or other proteins). Conversely, Hfq deletion will result in more CsgD and in an increase of amyloid cell content. This is the opposite of our observation. Thus, we believe that the changes observed in IR spectra are directly related to the level of Hfq expression, and not to other protein, which expression is dependent on Hfq.

**Figure 3:**
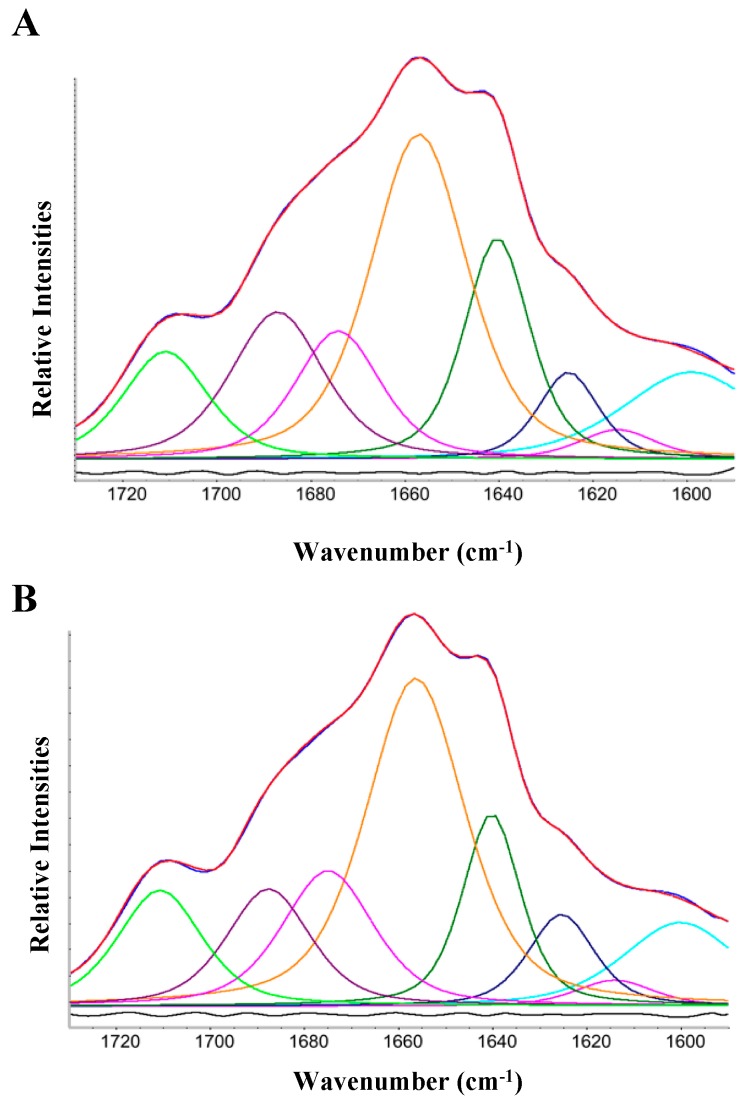
Curve fitting of Hfq full-length and Hfq-CTR (see also Table 1). Red: original spectrum, blue: composite spectrum, black: residual, other colors Gaussian/Lorentzian peaks. The residual was shifted for clarity (remark: the peak at 1600 cm^−1^ not assigned to protein secondary structure was not comprised in Table 1). **A**: full length Hfq; **B**: Hfq-CTR.

In Figure 3, we report a curve fitting procedure of the total amide I area, which gives us quantitative details in the secondary structure arrangements of full-length Hfq and Hfq devoid of its CTR in vivo (reported in Table 1). 

Both the full-length Hfq and the Hfq-CTR (devoid of NTR) difference spectra gave very similar curve fitting results with minor differences in the evaluation of the band positions and areas of a few percent. The β-sheet signal was separated into three peaks, one at 1614 cm^−1^ characteristic of protein aggregation showing that there was little aggregation in the bacteria, one at 1640 cm^−1^, assigned to normal β-sheets, and one at 1625 cm^−1^ assigned to amyloid β-sheet. In amyloids, a peak at ~ 1615 cm^−1^ can often be associated with a peak at ~1690 cm^−1^ (also observed here), and is attributed to intermolecular β-sheet contacts in mature fibrils and aggregates, while a ~1625 cm^−1^ can be attributed to intermolecular β-sheet contacts in smaller aggregates. In both in vivo analyses the amyloid β-sheet signal was approximately half of the β-sheet signal. Previous in vitro FTIR [24], and Circular Dichroism analyses (Supplementary Figure S1) indicated between 30 and 40% of β-sheet, in agreement with in vivo analysis. Note that amyloid fibrils formed in vitro or in vivo-isolated may, however, be structurally different [49]. While the peak at 1687 cm^−1^ suggests that a part of the protein adopts an antiparallel β-sheets conformation, the peak at ~1640 cm^−1^ may also be partially attributed to the presence of parallel β-sheet [50], a result confirmed by SRCD analysis (Supplementary Figure S1) using the BESTSEL algorithm [51,52]. The β-sheet specialized algorithm identified 16.6% and 13.8% of antiparallel and parallel β-sheet respectively (0.015% NMRSD).

### 2.2. Multivariate Analysis Results

**Figure 4:**
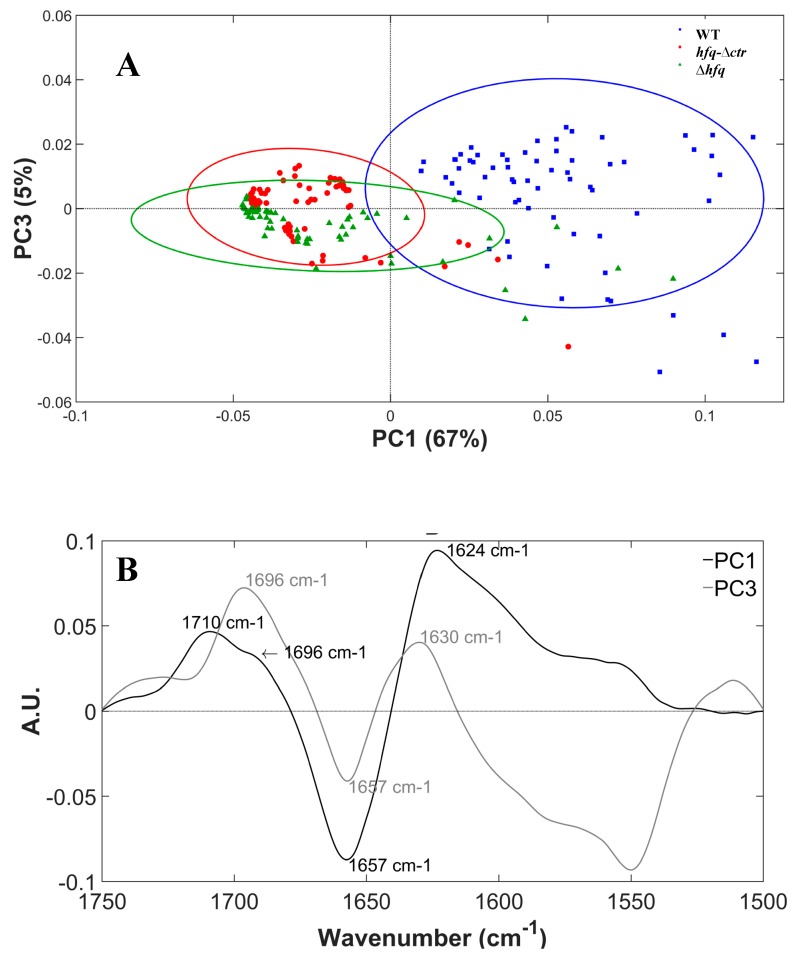
Principal Component Analysis of infrared spectra of the 3 strains. (**A**) PCA score plot with 95% confidence ellipses. Each point corresponds to a spectrum. The blue dots correspond to the strain expressing WT Hfq, the red dots to the strain expressing the NTR72-Hfq, and the green dots to the strain expressing no Hfq (∆*hfq*). We observed a near perfect separation of WT strain spectra from the other 2 strains on the PC1 axis. The ∆*hfq* and *hfq*∆*CTR* strains spectra not separated by the PC1 axis could be separated by the PC3 axis. (**B**) PCA loading plot for principal components 1 and 3 capturing respectively 67% and 5% of the spectral variance. PC1 loadings show positive peaks at 1710 and 1624 cm^−1^ that can be assigned to the presence of amyloid structures and are similar to that observed in the variance spectra in Supplementary Figure S2 PC3 loadings showing positive peaks at 1696 and 1630 cm^−1^ that can also be assigned to β-sheet structures.

Principal component analysis (PCA) is a multivariate statistical method for analyzing the variance of multidimensional sets of data, such as the variance in the FTIR spectra. Therefore, we performed a PCA on the spectra of the three *E. coli* strains to determine the variability between the different strains. The results of PCA in the amide I and II domain (1500–1750 cm^−1^) are shown in Figure 4. The score plot shows good separation between the WT strain and the two other strains along principal component 1 (PC1) axis while *hfq*∆*ctr* and ∆*hfq* strains could not be separated (Figure 4A). All WT strain spectra were classified on the positive side of the PC1 axis while only 5 *hfq*∆*ctr* strain and 7 ∆*hfq* strain spectra were positive along PC1 (6% and 10% of the spectra respectively). The loading plot from PC1 (Figure 4B) showed major positive peaks at 1624 and 1710 cm^−1^ with a strong shoulder at 1696 cm^−1^ and a negative peak at 1657 cm^−1^. The profile of PC1 was reminiscent of the difference spectra between WT and *hfq*∆*ctr* and WT and ∆*hfq* strains (Supplementary Figure S2). PCA loadings thus showed that WT strain spectra were differentiated from *hfq*∆*ctr* and ∆*hfq* strains spectra by their stronger absorption at 1710 and 1624 cm^−1^. The peak at 1624 cm^−1^ can be tentatively assigned to the peptide bonds involved in the β-sheet in the amyloid conformation. The peak at 1710 cm^−1^ was difficult to assign, but it could correspond to acidic amino acids that represent 13% of the CTR residues (see also Table 1). The shoulder at 1696 cm^−1^ could correspond to the antiparallel β-sheet conformation often found at 1690 cm^−1^ in proteins. PC1 carried 67% of the spectral variance and showed that the main spectral difference between the three strains was the formation of amyloid structures by the Hfq protein. All *hfq*∆*ctr* and ∆*hfq* strains spectra that clustered on the positive axis of PC1 were positioned on the negative side of the PC3 axis separated from the bulk of the WT strain spectra. PC3 showed major negative peaks at 1550 and 1657 cm^−1^ and positive peaks at 1630 and 1696 cm^−1^. This principal component seemed to capture a residual spectral variance related to the amyloid β−sheet conformation (1630 and 1696 cm^−1^) that was not captured in PC1, probably due to some residual baseline artifacts. It is possible that it also captured some signal from aggregated proteins that can be detected at around 1628 cm^−1^ in bacteria overexpressing exogenous genes [53]. However, PC3 contribution was small and represented only 5% of the spectral variance. Principal component 2 did not contribute to the separation between WT and *hfq*∆*ctr* and ∆*hfq* strains. It is likely that PC2 captured spectral variance linked to the typical variability of the proteome in a bacterial cell population with a positive peak at 1690 cm^−1^ and a negative peak at 1633 cm^−1^ that were not related to the expression of Hfq. The same signal could be observed when PCA was performed only on the ∆*hfq* spectra.

## 3. Materials and Methods 

### 3.1. E. coli Strains

*E. coli* BL21(DE3)Δ*hfq* strain was transformed with various plasmids allowing the expression of Hfq, i.e., full-length or NTR-72 (equivalent Hfq∆CTR) [54]. Plasmids were constructed using a QuickChange mutagenesis kit (Agilent Technologies, Santa Clara, CA), as previously described [24]. Our choice to use this strain was dictated by a tunable induction level, depending on the induction condition. As expected, the level of Hfq expressed was higher than that obtained by expression from the chromosomic copy. Nevertheless, we adapted our conditions (IPTG concentrations and induction time) to reduce the over-expression to approximately a factor of 5. Three strains expressing different forms of Hfq were tested and compared, namely the strain expressing Hfq full-length, CTR-truncated Hfq Hfq∆CTR = Hfq-NTR72 (residues 1 to 72 of Hfq) and no Hfq (empty plasmid = control strain). *E. coli* BL21(DE3)Δ*hfq* transformed strains were grown overnight in LB + ampicillin, diluted and further grown to an OD_600_ of 0.5. Hfq expression was turned on using isopropyl β-D-1-thiogalactopyranoside (IPTG) at 0.05 mM (various incubation time were tested, from 30 min to 3 h). Cells were collected by centrifugation and washed three times in water. Each culture was repeated at least three times (independent cultures). Note that Hfq protein devoid of its CTR is less stable than full-length Hfq and that it is less abundant when expressed in vivo [31]. As shown in sup Figure S3, in our condition we evaluate that expression of truncated Hfq is 30% less than that of full-length Hfq.

### 3.2. FTIRSpectroscopy and Principal Components Analysis

For infrared (IR) spectroscopy analysis, we acquired IR transmission spectra by depositing cells on a CaF_2_ surface. The deposits were then dried at room temperature and pressure. For each deposit, thirty spectra were acquired at different positions using a Thermo Scientific IN10 infrared microscope (Villebon sur Yvette, France) equipped with a DTGS detector, with a 15x Schwarzschild objective and using an aperture of 100 × 100 μm². Infrared absorption measurements were recorded with a resolution of 4 cm^−1^ and a zero filling factor of 2 in the region between 4000 and 400 cm^−1^ with 128 scans, but only the region between 1750 and 1500 cm^−1^ was analyzed and compared for the different strains. Spectra were first baseline-corrected and then normalized by unit vector normalization. Deconvolution of the bands has been performed with OMNIC software (Thermo Scientific, Villebon sur Yvette, France), using the method of the second derivative and curve fitting using a mixed Gaussian/Lorentzian shape. The 1590–1730 cm^−1^ range was fitted with eight to nine 20 cm^−1^-wide peaks with a total of 63 free parameters for 74 points in the spectral range.

Pre-processed and mean-centered spectra in the 1500–1750 cm^−1^ region were then subjected to Principal Component Analysis (PCA) using TheUnscrambler X 10.3 software (CAMO, Oslo, Norway) with the NIPALS (non-linear iterative partial alternating least square) algorithm and cross-validation (20 segments of 16 spectra). The PCA was performed with three to four principal components (PCs). The 95% confidence ellipses were computed in Matlab (Mathworks, Natick, MA) with an in-house script.

### 3.3. Synchrotron Radiation Circular Dichroism (SRCD)

For SRCD analysis, measurements and data collection were carried out on DISCO beamline at the SOLEIL Synchrotron (proposal #20180227) [55], as described previously [56]. Two to four microliter of each sample was loaded into circular demountable CaF_2_ cells (4.7-micron path length). Separated data collections were carried out to ensure repeatability. Spectral acquisitions of 1 nm steps at 1.2 s integration time, between 260 and 175 nm were performed in triplicate for the samples as well as for the baselines. (+)-camphor-10-sulfonic acid (CSA) was used to calibrate amplitudes and wavelength positions of the SRCD experiment. Data analyses including averaging, baseline subtraction, smoothing, scaling and standardization were carried out with CDtool [57]. The data-cutoff was at 175 nm based on the high tension (HT). Secondary structure content was determined using BestSel [51,52].

## 4. Conclusions

In this work, we confirm that the *E. coli* pleiotropic regulator Hfq forms an amyloid structure in vivo. Precisely, we show that this amyloid assembly is made by the Hfq CTR region and that Hfq NTR is not implicated in the formation of these structures. This is the first evidence for the presence of an amyloid structure inside bacteria using a label-free method. Indeed, FTIR can quantitatively and non-destructively detect amyloids in situ. One important outcome of this work would be to image the amyloid nanostructures inside the cell using nanoIR, a chemical imaging technique with <10 nm spatial resolution [58]. This should allow detection of amyloid folding in a cell as small as *E. coli* cell. Another continuation of this work would also be to analyze the kinetics of amyloid formation in live cells in different conditions (planktonic, biofilms or membrane stress for instance).

## Figures and Tables

**Table 1:** Hfq secondary structures content in vivo (in H_2_O at 20 °C) Note that acidic patch at the end of Hfq is not involved in amyloidogenesis nor in DNA binding properties (not shown).

Amide I Components (cm^−1^)	%	Assignment
**Hfq full-length in vivo**		
1614	2	intermolecular β-sheet
1625	5	amyloid β-sheet
1640	14.5	β-sheet
1656	31.5	random coil
1675	11	turns/β-aggregated
1687	13	β-sheet
1710	9	side chains
**Hfq-CTR in vivo**		
1614	2	intermolecular β-sheet
1625	6.5	amyloid β-sheet
1640	12	β-sheet
1656	34	random coil
1674	13	turns/β-aggregated
1687	10	β-sheet
1710	10	side chains

## Supplementary Materials

The following are available online at https://www.mdpi.com/2076-0817/8/1/36/s1, Figure S1: SRCD spectrum of Hfq-CTR in vitro. Figure S2: variance of spectra shown in Figure 1. Figure S3: Semi-quantitative Western Blot.

## References

[1] Munita J.M., Arias C.A. (2016). Mechanisms of Antibiotic Resistance. Microbiol. Spectr..

[2] Poole K. (2012). Stress responses as determinants of antimicrobial resistance in Gram-negative bacteria. Trends Microbiol..

[3] Zhang Y.F., Han K., Chandler C.E., Tjaden B., Ernst R.K., Lory S. (2017). Probing the sRNA regulatory landscape of *P. aeruginosa*: Post-transcriptional control of determinants of pathogenicity and antibiotic susceptibility. Mol. Microbiol..

[4] Mitarai N., Benjamin J.A., Krishna S., Semsey S., Csiszovszki Z., Masse E., Sneppen K. (2009). Dynamic features of gene expression control by small regulatory RNAs. Proc. Natl. Acad. Sci. USA.

[5] Papenfort K., Sun Y., Miyakoshi M., Vanderpool C.K., Vogel J. (2013). Small RNA-mediated activation of sugar phosphatase mRNA regulates glucose homeostasis. Cell.

[6] Salvail H., Masse E. (2012). Regulating iron storage and metabolism with RNA: An overview of posttranscriptional controls of intracellular iron homeostasis. Wiley Interdiscip. Rev. RNA.

[7] Sittka A., Pfeiffer V., Tedin K., Vogel J. (2007). The RNA chaperone Hfq is essential for the virulence of Salmonella typhimurium. Mol. Microbiol..

[8] Bardill J.P., Zhao X., Hammer B.K. (2011). The Vibrio cholerae quorum sensing response is mediated by Hfq-dependent sRNA/mRNA base pairing interactions. Mol. Microbiol..

[9] Chao Y., Vogel J. (2010). The role of Hfq in bacterial pathogens. Curr. Opin. Microbiol..

[10] Kendall M.M., Gruber C.C., Rasko D.A., Hughes D.T., Sperandio V. (2011). Hfq virulence regulation in enterohemorrhagic *Escherichia coli* O157:H7 strain 86-24. J. Bacteriol..

[11] Vogel J., Luisi B.F. (2011). Hfq and its constellation of RNA. Nat. Rev. Microbiol..

[12] Storz G., Vogel J., Wassarman K.M. (2011). Regulation by small RNAs in bacteria: Expanding frontiers. Mol. Cell.

[13] Majdalani N., Vanderpool C.K., Gottesman S. (2005). Bacterial small RNA regulators. Crit. Rev. Biochem. Mol. Biol..

[14] Aiba H. (2007). Mechanism of RNA silencing by Hfq-binding small RNAs. Curr. Opin. Microbiol..

[15] Hwang W., Arluison V., Hohng S. (2011). Dynamic competition of DsrA and rpoS fragments for the proximal binding site of Hfq as a means for efficient annealing. Nucleic Acids Res..

[16] Arluison V., Hohng S., Roy R., Pellegrini O., Regnier P., Ha T. (2007). Spectroscopic observation of RNA chaperone activities of Hfq in post-transcriptional regulation by a small non-coding RNA. Nucleic Acids Res..

[17] Battesti A., Majdalani N., Gottesman S. (2011). The RpoS-Mediated General Stress Response in *Escherichia coli* (*). Annu. Rev. Microbiol..

[18] Cayrol B., Fortas E., Martret C., Cech G., Kloska A., Caulet S., Barbet M., Trepout S., Marco S., Taghbalout A. (2015). Riboregulation of the bacterial actin-homolog MreB by DsrA small noncoding RNA. Integr. Biol. Quant. Biosci. Nano Macro.

[19] Mandin P., Gottesman S. (2010). Integrating anaerobic/aerobic sensing and the general stress response through the ArcZ small RNA. EMBO J..

[20] Guillier M., Gottesman S., Storz G. (2006). Modulating the outer membrane with small RNAs. Genes Dev..

[21] Vogt S.L., Raivio T.L. (2014). Hfq reduces envelope stress by controlling expression of envelope-localized proteins and protein complexes in enteropathogenic Escherichia coli. Mol. Microbiol..

[22] Feliciano J.R., Grilo A.M., Guerreiro S.I., Sousa S.A., Leitao J.H. (2016). Hfq: A multifaceted RNA chaperone involved in virulence. Future Microbiol..

[23] Diestra E., Cayrol B., Arluison V., Risco C. (2009). Cellular electron microscopy imaging reveals the localization of the Hfq protein close to the bacterial membrane. PLoS ONE.

[24] Fortas E., Piccirilli F., Malabirade A., Militello V., Trepout S., Marco S., Taghbalout A., Arluison V. (2015). New insight into the structure and function of Hfq C-terminus. Biosci. Rep..

[25] Taghbalout A., Yang Q., Arluison V. (2014). The *Escherichia coli* RNA processing and degradation machinery is compartmentalized within an organized cellular network. Biochem. J..

[26] Brennan R.G., Link T.M. (2007). Hfq structure, function and ligand binding. Curr. Opin. Microbiol..

[27] Wilusz C.J., Wilusz J. (2013). Lsm proteins and Hfq: Life at the 3′ end. RNA Biol..

[28] Partouche D., Malabirade A., Bizien T., Velez M., Trepout S., Marco S., Militello V., Sandt C., Wien F., Arluison V. (2018). Techniques to Analyze sRNA Protein Cofactor Self-Assembly In Vitro. Methods Mol. Biol..

[29] Malabirade A., Partouche D., El Hamoui O., Turbant F., Geinguenaud F., Recouvreux P., Bizien T., Busi F., Wien F., Arluison V. (2018). Revised role for Hfq bacterial regulator on DNA topology. Sci. Rep..

[30] Arluison V., Mura C., Guzman M.R., Liquier J., Pellegrini O., Gingery M., Regnier P., Marco S. (2006). Three-dimensional Structures of Fibrillar Sm Proteins: Hfq and Other Sm-like Proteins. J. Mol. Biol..

[31] Arluison V., Folichon M., Marco S., Derreumaux P., Pellegrini O., Seguin J., Hajnsdorf E., Regnier P. (2004). The C-terminal domain of *Escherichia coli* Hfq increases the stability of the hexamer. Eur. J. Biochem..

[32] Malabirade A., Morgado-Brajones J., Trepout S., Wien F., Marquez I., Seguin J., Marco S., Velez M., Arluison V. (2017). Membrane association of the bacterial riboregulator Hfq and functional perspectives. Sci. Rep..

[33] Malabirade A., Jiang K., Kubiak K., Diaz-Mendoza A., Liu F., van Kan J.A., Berret J.F., Arluison V., van der Maarel J.R.C. (2017). Compaction and condensation of DNA mediated by the C-terminal domain of Hfq. Nucleic Acids Res..

[34] Beich-Frandsen M., Vecerek B., Konarev P.V., Sjoblom B., Kloiber K., Hammerle H., Rajkowitsch L., Miles A.J., Kontaxis G., Wallace B.A. (2011). Structural insights into the dynamics and function of the C-terminus of the E. coli RNA chaperone Hfq. Nucleic Acids Res..

[35] Navarra G., Leone M., Militello V. (2007). Thermal aggregation of beta-lactoglobulin in presence of metal ions. Biophys. Chem..

[36] Navarra G., Troia F., Militello V., Leone M. (2013). Characterization of the nucleation process of lysozyme at physiological pH: Primary but not sole process. Biophys. Chem..

[37] Tatulian S.A. (2013). Structural characterization of membrane proteins and peptides by FTIR and ATR-FTIR spectroscopy. Methods Mol. Biol..

[38] Zandomeneghi G., Krebs M.R., McCammon M.G., Fandrich M. (2004). FTIR reveals structural differences between native beta-sheet proteins and amyloid fibrils. Protein Sci..

[39] Aguilera P., Marcoleta A., Lobos-Ruiz P., Arranz R., Valpuesta J.M., Monasterio O., Lagos R. (2016). Identification of Key Amino Acid Residues Modulating Intracellular and In vitro Microcin E492 Amyloid Formation. Front. Microbiol..

[40] Gasset-Rosa F., Coquel A.-S., Moreno-Del Alamo M., Chen P., Song X., Serrano A.M., Fernandez-Tresguerres M.E., Moreno-Diaz de la Espina S., Lindner A.B., Giraldo R. (2014). Direct assessment in bacteria of prionoid propagation and phenotype selection by Hsp70 chaperone. Mol. Microbiol..

[41] Vetri V., Carrotta R., Picone P., Di Carlo M., Militello V. (2010). Concanavalin A aggregation and toxicity on cell cultures. Biochim. Biophys. Acta.

[42] Stains C.I., Mondal K., Ghosh I. (2007). Molecules that target beta-amyloid. ChemMedChem.

[43] D’Amico M., Di Carlo M.G., Groenning M., Militello V., Vetri V., Leone M. (2012). Thioflavin T Promotes Aβ(1−40) Amyloid Fibrils Formation. Biophys. Chem..

[44] Zhou Y., Blanco L.P., Smith D.R., Chapman M.R. (2012). Bacterial amyloids. Methods Mol. Biol..

[45] Zambrano N., Guichard P.P., Bi Y., Cayrol B., Marco S., Arluison V. (2009). Involvement of HFq protein in the post-transcriptional regulation of E. coli bacterial cytoskeleton and cell division proteins. Cell Cycle.

[46] Balasubramanian D., Ragunathan P.T., Fei J., Vanderpool C.K. (2016). A Prophage-Encoded Small RNA Controls Metabolism and Cell Division in Escherichia coli. mSystems.

[47] Serra D.O., Mika F., Richter A.M., Hengge R. (2016). The green tea polyphenol EGCG inhibits E. coli biofilm formation by impairing amyloid curli fibre assembly and downregulating the biofilm regulator CsgD via the sigma(E)-dependent sRNA RybB. Mol. Microbiol..

[48] Andreassen P.R., Pettersen J.S., Szczerba M., Valentin-Hansen P., Moller-Jensen J., Jorgensen M.G. (2018). sRNA-dependent control of curli biosynthesis in Escherichia coli: McaS directs endonucleolytic cleavage of csgD mRNA. Nucleic Acids Res..

[49] Terry C., Harniman R.L., Sells J., Wenborn A., Joiner S., Saibil H.R., Miles M.J., Collinge J., Wadsworth J.D.F. (2019). Structural features distinguishing infectious ex vivo mammalian prions from non-infectious fibrillar assemblies generated in vitro. Sci. Rep..

[50] Chirgadze Y.N., Nevskaya N.A. (1976). Infrared spectra and resonance interaction of amide-I vibration of the paraellel-chain pleated sheets. Biopolymers.

[51] Micsonai A., Wien F., Kernya L., Lee Y.H., Goto Y., Refregiers M., Kardos J. (2015). Accurate secondary structure prediction and fold recognition for circular dichroism spectroscopy. Proc. Natl. Acad. Sci. USA.

[52] Micsonai A., Wien F., Bulyaki E., Kun J., Moussong E., Lee Y.H., Goto Y., Refregiers M., Kardos J. (2018). BeStSel: A web server for accurate protein secondary structure prediction and fold recognition from the circular dichroism spectra. Nucleic Acids Res..

[53] Ami D., Bonecchi L., Calì S. (2003). FT-IR study of heterologous protein expression in recombinant *Escherichia coli* strains. Biochim. Biophys. Acta.

[54] Jiang K., Zhang C., Guttula D., Liu F., van Kan J.A., Lavelle C., Kubiak K., Malabirade A., Lapp A., Arluison V. (2015). Effects of Hfq on the conformation and compaction of DNA. Nucleic Acids Res..

[55] Refregiers M., Wien F., Ta H.P., Premvardhan L., Bac S., Jamme F., Rouam V., Lagarde B., Polack F., Giorgetta J.L. (2012). DISCO synchrotron-radiation circular-dichroism endstation at SOLEIL. J. Synchrotron Radiat..

[56] Partouche D., Turbant F., El Hamoui O., Campidelli C., Bombled M., Trepout S., Wien F., Arluison V. (2018). Epigallocatechin Gallate Remodelling of Hfq Amyloid-Like Region Affects *Escherichia coli* Survival. Pathogens.

[57] Lees J.G., Smith B.R., Wien F., Miles A.J., Wallace B.A. (2004). CDtool-an integrated software package for circular dichroism spectroscopic data processing, analysis, and archiving. Anal. Biochem..

[58] Dazzi A., Prater C.B. (2016). AFM-IR: Technology and Applications in Nanoscale Infrared Spectroscopy and Chemical Imaging. Chem. Rev..

