# Supplementary figures and images for: In Situ Characterization of Hfq Bacterial Amyloid: A Fourier-Transform Infrared Spectroscopy Study
Source: Pathogens. 2019 Mar 18;8(1):36. doi: 10.3390/pathogens8010036 (PMC6471401; doi:10.3390/pathogens8010036)

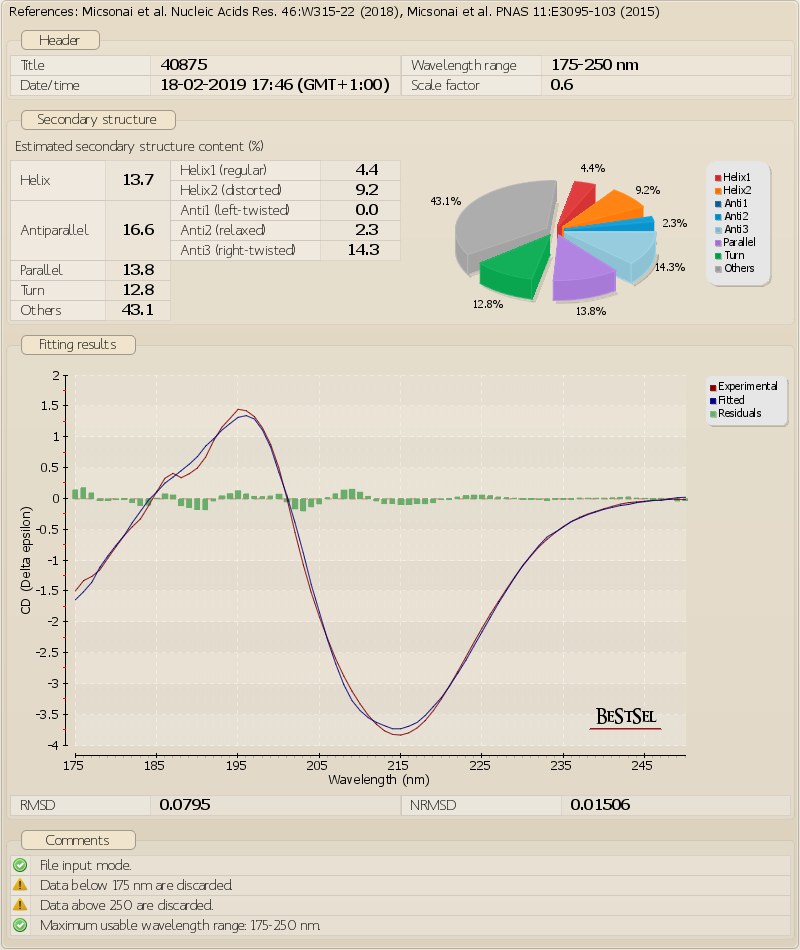

Supplement: Supplementary file 1 [file pathogens-08-00036-s001.zip › supfigurepartouche/SupFigS1.png]

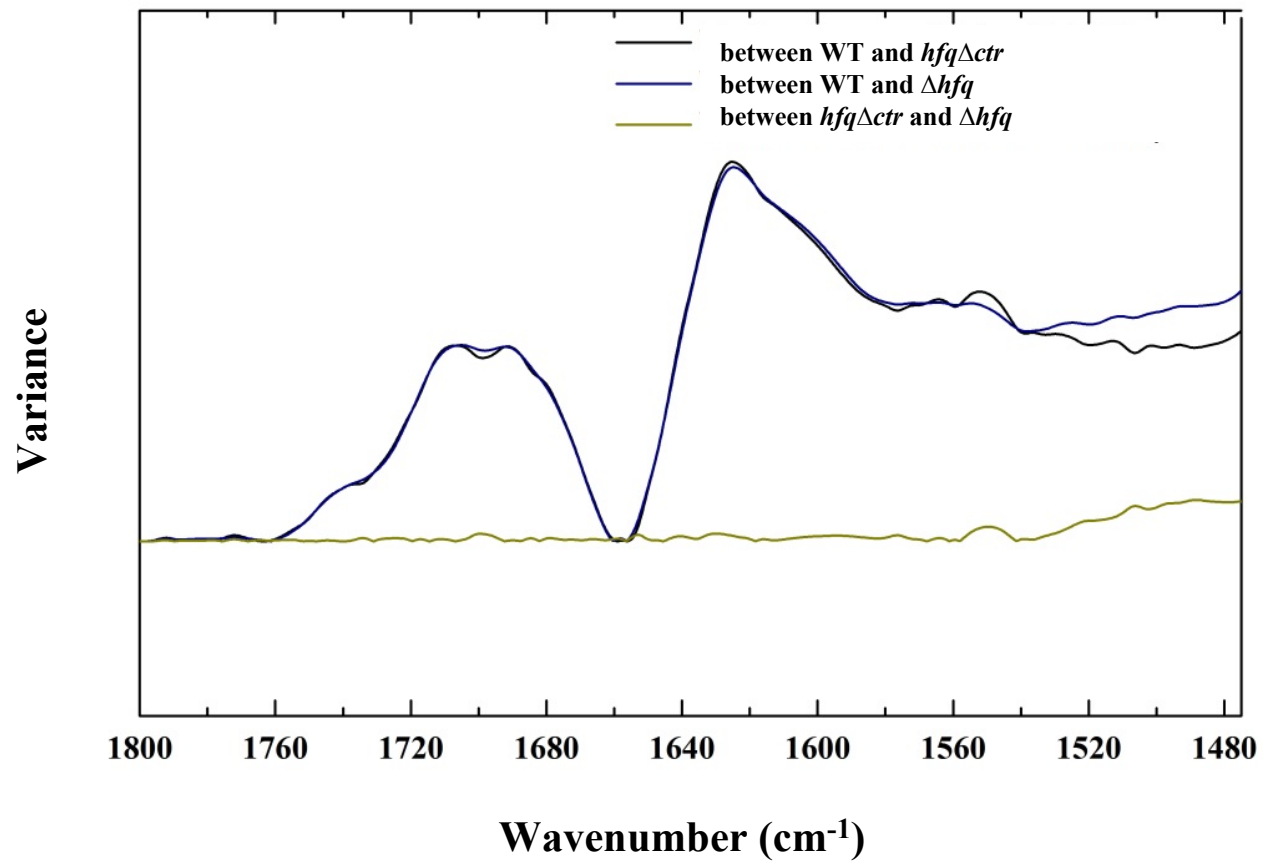

Supplement: Supplementary file 1 [file pathogens-08-00036-s001.zip › supfigurepartouche/SupfigS2.pdf]

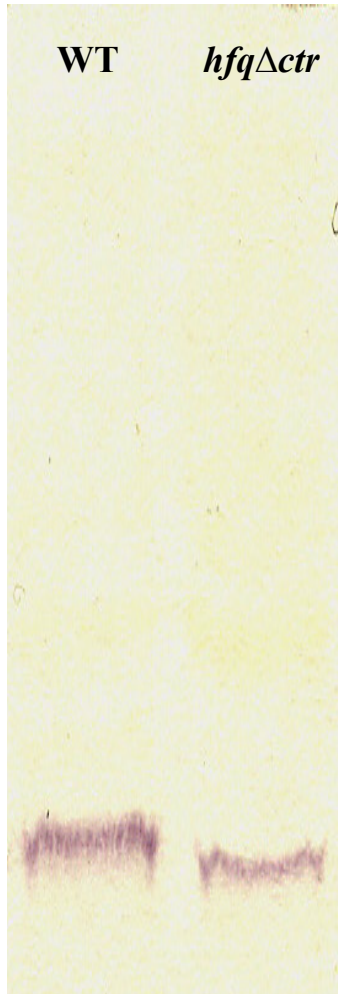

**Sup. Fig. S3:** Western blot were performed as described in (23)

Supplement: Supplementary file 1 [file pathogens-08-00036-s001.zip › supfigurepartouche/SupFigS3.pdf]
